# Effect of Local Injection of Lidocaine and Articaine plus Epinephrine on Methemoglobin Level during General Anesthesia

**DOI:** 10.30476/DENTJODS.2021.88011.1306

**Published:** 2022-03

**Authors:** Hassan Mohajerani, Fatemeh Latifi, Reza Tabrizi, Shervin Shafiei, Hamidreza Moslemi, Maryam Ebadi

**Affiliations:** 1 Dept. of Oral and Maxillofacial Surgery, Dental School, Shahid Beheshti University of Medical Sciences, Tehran, Iran; 2 Postgraduate student, Dept. of Oral and Maxillofacial Surgery, Dental School, Shahid Beheshti University of Medical Sciences, Tehran, Iran; 3 Dentist, Shiraz, Iran

**Keywords:** Methemoglobinemia, Local anesthesia, General Anesthesia, Methemoglobin

## Abstract

**Statement of the Problem:**

Methemoglobinemia is a potentially life-threatening rare medical condition, which refers to an increase in the level of oxidized form of hemoglobin (methemoglobin). Excessive replacement of hemoglobin with methemoglobin leads to functional hypoxia and even fatal conditions.

**Purpose:**

The aim of this study was to evaluate the effect of two common local anesthetic agents namely lidocaine and articaine administered for hemostasis during surgery on methemoglobin level.

**Materials and Method:**

This prospective cohort study was conducted from January 2017 to December 2019. Demographic data including age, gender, and weight of patients were collected. Sixty patients were randomly divided into three groups (n=20) regarding the local anesthetic agent administered for hemostasis during surgery as lidocaine group (group 1), articaine group (group 2), and control group (no local anesthetic; group 3). The patients were candidates for orthognathic surgery, reconstruction of the maxillary and mandibular atrophic ridges with autogenous grafts, and reconstruction of maxillofacial fractures. The methemoglobin level was measured before surgery and six hours after the initiation of surgery.

**Results:**

The mean age and weight of patients were not significantly different among the three groups (*p*= 0.891 and *p*= 0.416, respectively). No significant differences were observed among the three groups regarding the gender distribution (*p*= 0.343) or type of surgery (p= 0.990). Statistical analysis did not show any significant difference in the mean baseline methemoglobin level among the three groups (*p*= 0.109). Although the mean methemoglobin values increased in the three groups, paired sample t-test did not show any significant change in the values at six hours after the initiation of surgery compared with baseline in any of the three groups (*p*= 0.083 for group 1, *p*= 0.096 for group 2, and *p*= 0.104 for group 3).

**Conclusion:**

The results demonstrated that administration of lidocaine and articaine plus epinephrine for hemostasis during general anesthesia are equally safe.

## Introduction

Methemoglobinemia is a rare medical condition, which is potentially life threatening especially when it is not recognized and treated early [ 1 ]. Methemoglobinemia refers to an increase in the level of oxidized form of hemoglobin (methemoglobin). It may be either congenital due to an abnormality in the hemoglobin structure or methemoglobin reductase enzyme, or acquired secondary to consumption of chemicals and drugs that oxidize hemoglobin [ 1 - 3 ]. Moreover, there are some reports regarding methemoglobinemia following conditions such as gastrointestinal infections, sepsis, and sickle cell crisis, which are highly uncommon conditions [ 1 - 3 ].

The oxidized form of hemoglobin (ferric form) cannot properly bind to oxygen, and excessive replacement of hemoglobin with methemoglobin leads to functional hypoxia [ 4 ]. The upper normal limit of methemoglobin is 1.5% and when it rises, patients develop a variety of signs and symptoms from mild to severe and life-threatening conditions. Mild conditions are accompanied by cyanosis, dizziness, shortness of breath, cough, low blood oxygen saturation, and brown color arterial blood samples [ 5 - 7 ]. When the methemoglobin level exceeds 55%, patients experience unconsciousness and lethargy. Higher levels of methemoglobin (>70%) cause neurological and cardiac failure and could be fatal [ 5 - 7 ]. 

As mentioned earlier, acquired methemoglobinemia occurs in some patients following their exposure to some chemicals or drugs. Local anesthetic agents are among the medications that contribute to methemoglobinemia when administered for local anesthesia in conscious patients or for regional blocks and hemostasis during general anesthesia [ 2 - 3 , 8 ]. Local anesthetic agents in dentistry and oral and maxillofacial surgery are administered both for local anesthesia and hemostasis. A review of the published reports regarding methemoglobinemia related to local anesthetic agents named benzocaine, prilocaine, and lidocaine as the top three local anesthetic agents causing methemoglobinemia with a prevalence of 66%, 28% and 5%, respectively. Data also demonstrated that most of the cases occurred outside of the operating room [ 3 , 8 ]. There are much fewer cases regarding articaine and methemoglobinemia [ 1 ]. Lidocaine and articaine plus epinephrine are two common local anesthetic agents administered for hemostasis during surgery. Because of the uncommon nature of methemoglobinemia and the need for its early management, early detection of this condition and the potential risk of its development are imperative by dentists or surgeons who administer oxidizing agents. 

The aim of this study was to evaluate the effect of these two agents on methemoglobin level.

## Materials and Method

This prospective cohort study was conducted in the Department of Oral and Maxillofacial Surgery of Taleghani Hospital between January 2017 and December 2019. Patients >12 years of age that weighed >32 Kg, had no systemic disease (ASA I and II), and were candidates for orthognathic surgery, reconstruction of the maxillary and mandibular atrophic ridges with autogenous grafts, and reconstruction of maxillofacial fractures were included in this study. The exclusion criteria were congenital methemoglobinemia, pre-existing coronary artery disease, peripheral vascular disease, cerebrovascular diseases, sepsis, respiratory diseases, acidosis, anemia and glucose-6-phosphate dehydrogenase deficiency. 

The objective and design of the study were completely explained to all participants and they were requested to read and sign the informed consent form. This study was performed according to the principles outlined by the World Medical Association’s Declaration of Helsinki on experimentations involving human subjects, as revised in 2000. The protocol of the study was also approved by the Ethics Committee of Shahid Beheshti University of Medical Sciences (code: IR. SBMU.RIDS.REC.1395.248). Demographic data including age, gender and weight of patients were collected in a data collection form at the time of admission. The patients were randomly divided into three groups based on the type of local anesthetic agent used for hemostasis during surgery. Group 1 consisted of patients for whom, 20mg/mL lidocaine hydrochloride plus 0.0125 mg/mL epinephrine (Persocaine E; Darou Pakhsh®, Tehran, Iran) was administered. Patients in group 2 received 40mg/mL articaine hydrochloride plus 0.005 mg/mL epinephrine (Dentacain; Exir®, Tehran, Iran). Group 3 was the control group and received no local anesthetic agent. The patients’ methemoglobin levels were measured using a pulse oximeter (Masimo Radical-7® Pulse CO-Oximeter®). The methemoglobin value was measured before surgery, and then all patients underwent general anesthesia with a similar protocol. Six hours after the initiation of surgery, the level of methemoglobin was measured again to determine the effect of the administered local anesthetic agent on the level of methemoglobin. 

Statistical analyses were performed using SPSS version 23 (SPSS Inc., IL, USA). One-way ANOVA was used to compare the mean age, weight, and baseline methemoglobin value among the three groups. The Chi-square test was used to compare gender distribution and type of surgery among the three groups. Paired sample t-test was used to compare methemoglobin changes during the study period in the three groups. The *p* Values <0.05 were considered statistically significant.

## Results

Sixty patients in three groups were evaluated in this study (n=20 in each group). Table 1 presents the mean age and weight of patients. Statistical analysis showed no significant difference regarding age and weight among the three groups (*p*= 0.891 and *p*= 0.416, respectively). There were 8 males and 12 females in group 1, and groups 2 and 3 consisted of 12 males and 8 females. No significant differences were observed among the three groups in gender distribution (*p*= 0.343). Table 2 shows the distribution of type of surgery among the three groups. There was no significant difference regarding type of surgery among the three groups either (*p*=0.990).

**Table 1:** Mean age and weight of patients in lidocaine, articaine and control groups

Group	Age			Weight		
	Mean	SD	*p* Value	Mean	SD	*p* Value
Group 1	35.30	12.13	0.891	80.20	10.92	0.416
Group 2	36.35	12.93		78.70	12.29	
Group 3	37.30	14.34		74.00	20.84	

Group 1= Lidocaine group, Group 2= Articaine group, Group 3= Control group (no anesthetic agent), SD= Standard deviation

**Table 2:** Distribution of type of surgery in lidocaine, articaine, and control groups

	Surgery type (n)				*p*Value
	Orthognathic surgery	Reconstruction of maxillofacial fractures	Reconstruction of atrophic ridges with autogenous graft	Total	
Group 1	7	7	6	20	0.990
Group 2	7	6	7	20	
Group 3	8	6	6	20	

Group1= Lidocaine group, Group 2= Articaine group, Group 3= Control group (no anesthetic agent)

The baseline methemoglobin level was compared among the three groups. The mean baseline methemoglobin level (± standard deviation) was 0.42±0.14% in group 1, 0.48±0.11% in group 2, and 0.39±0.13% in group 3. No statistically significant difference was observed among the three groups in this respect (*p*= 0.109). Table 3 shows the changes in methemoglobin level in the three groups. Although the mean methemoglobin values increased in the three groups, paired sample t-test did not show any significant change in values at six hours after the initiation of surgery compared with baseline in any of the three groups (*p*=0.083 for group 1, *p*= 0.096 for group 2, and *p*= 0.104 for group 3).

**Table 3:** Methemoglobin alterations in the lidocaine, articaine and control groups

	Methemoglobin values				*p* Value
	Baseline		Six hours after the initiation of surgery		
	Mean	SD	Mean	SD	
Group 1	0.42	0.14	0.43	0.13	0.083
Group 2	0.48	0.11	0.49	0.11	0.096
Group 3	0.39	0.13	0.40	0.12	0.104

Group 1= Lidocaine group, Group 2= Articaine group, Group 3= Control group (no anesthetic agent), SD= Standard deviation

## Discussion

High levels of methemoglobin are usually well-tolerated by healthy individuals, but very high concentrations or concurrence with compromised cardiopulmonary reserve can lead to rapid toxicity [ 1 , 4 ]. The acquired methemoglobinemia is much more common than the hereditary type, and occurs after exposure to some certain chemicals or medications in some patients. The medications and chemical agents with the potential to cause methemoglobinemia are as follows: nitrite derivatives such as nitric oxide and amyl nitrite, nitrate derivatives such as nitroglycerin, sulfonamides, phenacetin and phenazopyridine, some local anesthetic agents such as prilocaine, topical anesthetic agents such as lidocaine, prilocaine, benzocaine and antimalarial agents [ 1 , 4 ].

A review of recent studies regarding the prevalence of methemoglobinemia following the administration of local and topical anesthetic agents and a comparison between them demonstrated that data are heterogeneous, and most available studies on this topic are case reports. Alanazi *et al*. [ 1 ], in their literature review reported that prilocaine had higher potential to cause methemoglobinemia due to its otoluidine and aniline-like structure and metabolism compared with other local anesthetic agents. Different predictive factors could affect the methemoglobin rise following the administration of prilocaine and other local anesthetic agents. For instance, Vasters *et al*. [ 9 ] suggested that a higher dose of prilocaine, female sex, and younger age were associated with a higher increase in the level of methemoglobin in patients after peripheral nerve block by prilocaine. Benzocaine is another anesthetic agent that has been repeatedly mentioned in case reports regarding methemoglobinemia.

The FDA report in 2013 reported 375 cases of methemoglobinemia following benzocaine administration (eight fatal reports) and only 16 cases following lidocaine administration. Recent studies reported that topical benzocaine application contributed to methemoglobinemia in patients after transesophageal echocardiog raphy and endoscopic and dental procedures and caused a higher rate of methemoglobinemia compared with lidocaine [ 10 - 13 ].

In our study, we evaluated the top two most commonly used local anesthetic agents with epinephrine that are used for local infiltration anesthesia for both anesthetic and hemostatic propose. As mentioned earlier, no significant rise in the methemoglobin level occurred after the local administration of anesthetic agents. These results demonstrated that lidocaine and articaine were the same regarding their effect on the methemoglobin level, and were equally safe for administration during general anesthesia. There are much fewer reports and studies about articaine in the recent literature compared with lidocaine. A review article presented by Alanazi *et al*. [ 1 ] highlighted the importance of the administration route of articaine. According to their results, articaine did not increase the methemoglobin level when administered for dental anesthesia, but methemoglobinemia can occur following intravenous regional anesthesia by articaine. Reports regarding methemoglobinemia following administration of lidocaine in dental procedures, transesophageal echocardiography, endoscopy, and endobronchial intubation are noticeable in the literature, but there is a general agreement regarding the clinically tolerable methemoglobin levels following lidocaine administration [ 2 , 5 , 14 ]. 

## Conclusion

Within the limitations of this study, the results demonstrated that administration of lidocaine or articaine plus epinephrine for hemostasis in oral and maxillofacial surgeries was not associated with any increase in methemoglobin level and these agents are equally safe for administration during general anesthesia.

## Conflict of Interest

None 
